# Supplementary material for: Polygenic risk scores in cardiovascular risk prediction: A cohort study and modelling analyses
Source: PLoS Med. 2021 Jan 14;18(1):e1003498. doi: 10.1371/journal.pmed.1003498 (PMC7808664; doi:10.1371/journal.pmed.1003498)
Supplement: S1 Analysis Plan — (DOCX) [file pmed.1003498.s001.docx]

**UK Biobank Genetic CVD risk prediction projects**

***1. Key question (highest priority, flagged for rapid completion): What is the incremental predictive value of a polygenic risk score for CVD risk assessment?***

*Main aim*

To quantify the incremental predictive value of assessing polygenic risk score (PRS) for CVD risk prediction based on models with increasing number of conventional risk factors.

*Population of interest*

Participants in UK Biobank without a known history of CVD at entry into the study (ie, CHD, stroke, transient ischemic attack, peripheral vascular disease, cardiovascular surgery, pulmonary heart diseases, atrial fibrillation, or heart failure)

*Outcome of interest*

Primary outcome: CVD (ie, a combination of CHD [defined as fatal or non-fatal myocardial infarction and CHD] and stroke). Secondary outcomes: component CVD outcomes separately (ie, CHD and stroke). Participants will contribute only the first CVD outcome (whether nonfatal or fatal) recorded during follow-up (ie, deaths preceded by nonfatal CVD events will not be included).

*“Baseline” CVD risk prediction algorithm*

We will assess the incremental predictive value of PRS against “baseline” models with increasing number of conventional CVD risk factors. Information included in the “baseline” model will include: age, sex, smoking status, systolic blood pressure, history of diabetes, total and HDL-cholesterol (ie, “conventional” risk factors). Secondary analyses will include other CVD risk factors considered in CVD risk prediction (e.g. ethnicity, treatment for high blood pressure, novel lipids, inflammation biomarkers, glycaemia biomarkers, and kidney function biomarkers, among others).

*Polygenic risk score*

We will use as primary exposure variable a PRS specifically developed to predict CVD as a composite outcome. Secondarily, we will consider the simultaneous inclusion of PRSs developed to predict CHD and stroke separately. The PRSs will be developed using consistent methodology that combines all (external) relevant sets of GWAS summary statistics using a meta-scoring framework. UK Biobank data used in development of PRSs will be excluded from the evaluations.

*Analytical approach*

We will employ measures of discrimination (C-index, Royston’s D), and re-classification (eg, net reclassification improvement and integrated discrimination improvement) using stratified Cox’s models. We will examine categorical net reclassification of participants across predicted risk categories using cut-offs defined by the American College of Cardiology (ACC) and American Heart Association (AHA) 2013 (ie, <5%, 5% to <7·5%, and ≥7·5%), and other guidelines. To assess the clinical implications of adding PRS, we will estimate the number of individuals who would be eligible for treatment and the potential CVD events avoided (ie, number needed to screen).

We will compare the incremental risk prediction afforded by PRS on top of that provided by conventional risk factors commonly used in risk algorithms (eg, total and HDL cholesterol) as well as to that provided by other blood-based biomarkers available in UK Biobank (eg, C-reactive protein, HbA1c, ApoB).
