# Supplementary figures and images for: Polygenic risk scores in cardiovascular risk prediction: A cohort study and modelling analyses
Source: PLoS Med. 2021 Jan 14;18(1):e1003498. doi: 10.1371/journal.pmed.1003498 (PMC7808664; doi:10.1371/journal.pmed.1003498)

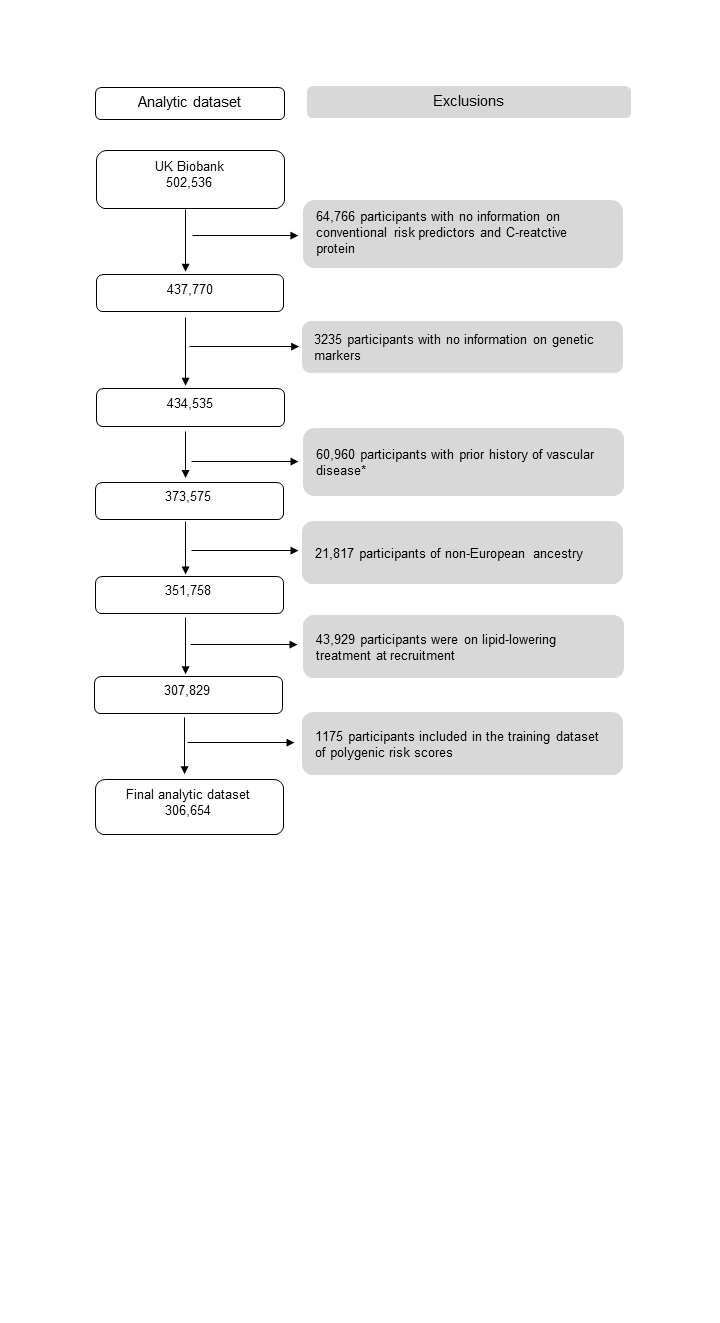

Supplement: S1 Fig — *Prior history of cardiovascular disease at baseline included coronary heart disease, angina, other heart disease, stroke, transient ischaemic attack, peripheral vascular disease, and cardiac revascularisations. (TIF) [file pmed.1003498.s002.tif]

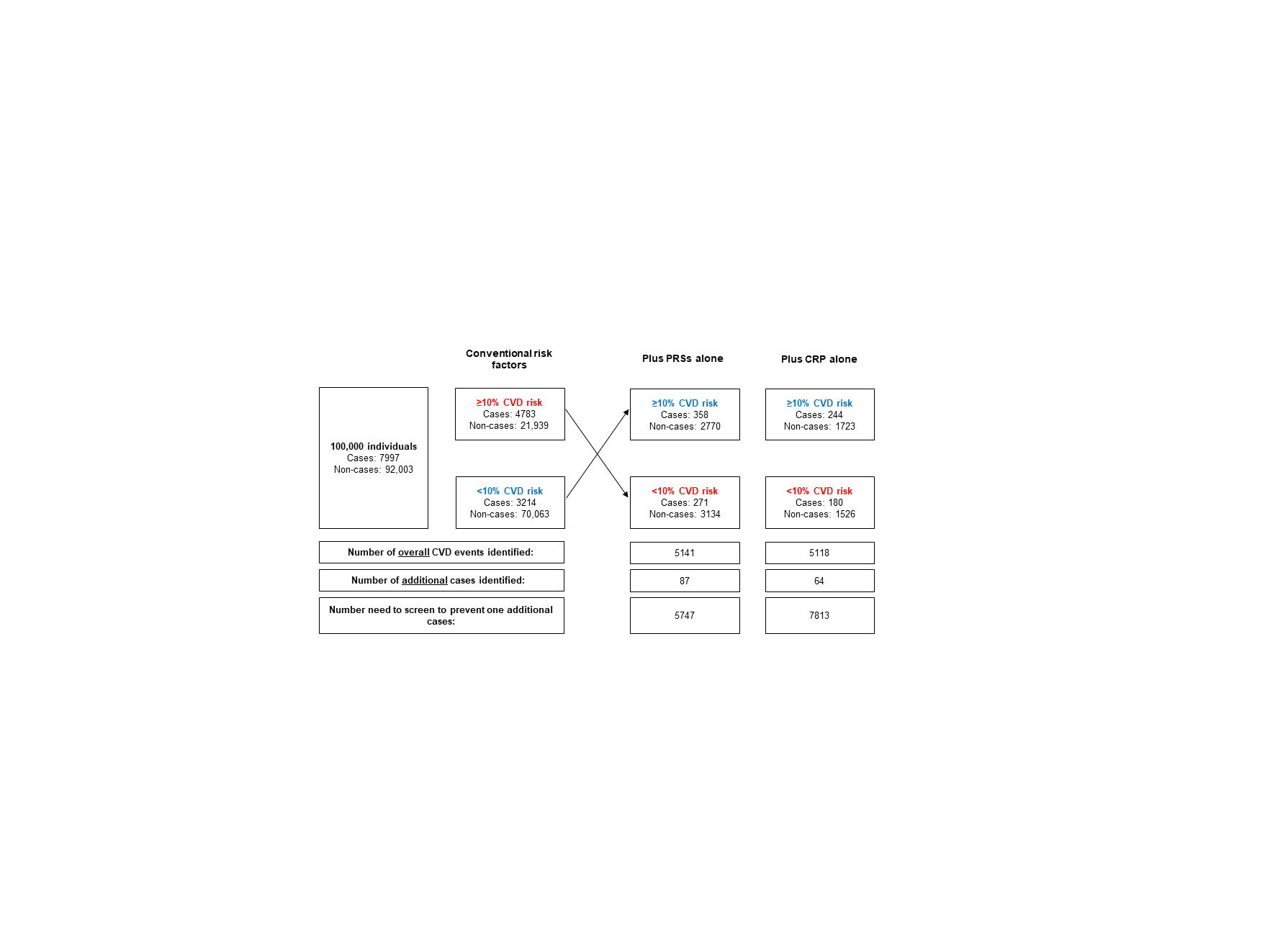

Supplement: S13 Fig — Numbers in red are shown for individuals who were initially classified as being at high risk and were reclassified down to intermediate risk; numbers in blue are shown for individuals moving from intermediate risk to high risk. Among 100,000 individuals, 1,197 cases and 7,354 non-cases were treated, irrespective of their 10-year CVD risk, since they had history of diabetes or LDL cholesterol ≥ 5.0 mmol/l. The number of cases screened as high risk, or classified as high risk due to diabetes or LDL cholesterol level, using conventional risk factors alone was 5,054, and thus, the number of events prevented was 1,011 (5,054 × 0.2). (TIF) [file pmed.1003498.s014.tif]
