# Supplementary material for: Polygenic risk scores in cardiovascular risk prediction: A cohort study and modelling analyses
Source: PLoS Med. 2021 Jan 14;18(1):e1003498. doi: 10.1371/journal.pmed.1003498 (PMC7808664; doi:10.1371/journal.pmed.1003498)
Supplement: S12 Fig — (DOCX) [file pmed.1003498.s013.docx]

| Per 100,000 screened | |
| --- | --- |
| Cases | Non-cases |
| 7997 | 92,003 |

Stratification of 10-year CVD risk according to conventional risk factors

| <5.0% CVD risk | |  | 5 – 7.5% CVD risk | |  | ≥ 7.5% CVD risk | |
| --- | --- | --- | --- | --- | --- | --- | --- |
| 46,603 | |  | 15,891 | |  | 37,506 | |
| Cases | Non-cases |  | Cases | Non-cases |  | Cases | Non-cases |
| 1143 | 45,460 |  | 1003 | 14,888 |  | 5851 | 31,655 |

Treatment assumed

Individuals would be recommended to start treatment if they have (n=1407):

- Diabetes
- LDL-C ≥ 5.0 mmol/L

| 5 – 7.5% CVD risk | |
| --- | --- |
| 14,484 | |
| Cases | Non-cases |
| 905 | 13,579 |

Reclassification of 10-year CVD risk with additional assessment of PRSs

| 0 – 5% CVD risk | |  | 5 – 7.5% CVD risk | |  | ≥ 7.5% CVD risk | |
| --- | --- | --- | --- | --- | --- | --- | --- |
| 4018 | |  | 7501 | |  | 2965 | |
| Cases | Non-cases |  | Cases | Non-cases |  | Cases | Non-cases |
| 185 | 3833 |  | 471 | 7030 |  | 249 | 2716 |
