# Supplementary material for: Polygenic risk scores in cardiovascular risk prediction: A cohort study and modelling analyses
Source: PLoS Med. 2021 Jan 14;18(1):e1003498. doi: 10.1371/journal.pmed.1003498 (PMC7808664; doi:10.1371/journal.pmed.1003498)
Supplement: S1 Table — (DOCX) [file pmed.1003498.s015.docx]

**S1 Table. Definition of study outcomes**

|  | **ICD-10 codes** | **OPCS-4 codes** |
| --- | --- | --- |
| ***Primary outcome*** |  |  |
| Cardiovascular disease (CVD) | I21-I23; fatal I24-I25, I60-69 |  |
| ***Secondary outcomes*** |  |  |
| Coronary heart disease | I21-I23; fatal I24-I25 |  |
| Stroke | I60-69 |  |
| CVD and cardiac revascularisations | I21-I23; fatal I24-I25; I60-69 | K40-K46, K49, K50.1, 50.2, K50.4, or K75 |
