# Supplementary material for: Polygenic risk scores in cardiovascular risk prediction: A cohort study and modelling analyses
Source: PLoS Med. 2021 Jan 14;18(1):e1003498. doi: 10.1371/journal.pmed.1003498 (PMC7808664; doi:10.1371/journal.pmed.1003498)
Supplement: S2 Table — Conventional risk factors included age at baseline, sex, smoking status, history of diabetes, systolic blood pressure, total cholesterol, and HDL cholesterol. Prediction model was developed using Cox regression for all participants, stratified by study centre and sex, adjusted for conventional risk predictors, where appropriate. Polygenic risk scores included the polygenic risk score for CHD and the one for ischaemic stroke (see Fig 2) as 2 linear predictors in the model throughout. (DOCX) [file pmed.1003498.s016.docx]

| **S2 Table. Incremental predictive ability of polygenic risk scores, and C-reactive protein, above conventional risk factors, by population characteristics at baseline** | | | | | | | | | | |
| --- | --- | --- | --- | --- | --- | --- | --- | --- | --- | --- |
| **Subgroup** | **No. of** | | | **C-index** | | | **C-index changes vs. Reference model** | | | |
|  | **Events / Total** | | | **Reference model** | | | **+ PRSs only** | | **+ CRP only** | |
| **Sex** | | | | | | | | | | |
| Male | 3764 / 131,881 | | | 0.6985 (0.6902, 0.7068) | | | 0.0182 (0.0145, 0.0220) | | 0.0052 (0.0031, 0.0074) | |
| Female | 1916 / 174,773 | | | 0.7260 (0.7141, 0.7379) | | | 0.0051 (0.0019, 0.0082) | | 0.0028 (0.0005, 0.0051) | |
|  |  | | |  | | | χ_1_=27.8; P_heter_<0.001 | | χ_1_=2.2; P_heter_ =0.14 | |
| **Age at baseline, years** |  |  | |  |  | |  |  |  |  |
| <55 | 1330 / 134,614 | | | 0.7030 (0.6879, 0.7181) | | | 0.0150 (0.0073, 0.0228) | | 0.0046 (-0.0006, 0.0098) | |
| 55-<65 | 2647 / 126,289 | | | 0.6461 (0.6346, 0.6576) | | | 0.0159 (0.0104, 0.0214) | | 0.0070 (0.0028, 0.0112) | |
| >=65 | 1703 / 45,751 | | | 0.6143 (0.5998, 0.6287) | | | 0.0229 (0.0148, 0.0309) | | 0.0035 (0.0007, 0.0063) | |
|  |  | | |  | | | χ_2_=2.4; P_heter_ =0.30 | | χ_2_=1.9; P_heter_ =0.39 | |
| **History of diabetes** | | | | | | | | | | |
| No | 5516 / 303,015 | | | 0.7084 (0.7013, 0.7154) | | | 0.0121 (0.0094, 0.0148) | | 0.0044 (0.0027, 0.0061) | |
| Yes | 164 / 3639 | | | 0.6734 (0.6314, 0.7155) | | | 0.0118 (-0.0014, 0.0249) | | 0.0030 (-0.0020, 0.0080) | |
|  |  | | |  | | | χ_1_=0.1; P_heter_ =0.96 | | χ_1_=0.3; P_heter_ =0.59 | |
| **Smoking status** | | | | | | | | | | |
| Not current | 4513 / 275,105 | | | 0.6991 (0.6913, 0.7070) | | | 0.0124 (0.0094, 0.0155) | | 0.0042 (0.0024, 0.0060) | |
| Current | 1167 / 31, 549 | | | 0.6848 (0.6699, 0.6997) | | | 0.0153 (0.0088, 0.0217) | | 0.0079 (0.0033, 0.0125) | |
|  |  | | |  | | | χ_1_=0.6; P_heter_ =0.44 | | χ_1_=2.2; P_heter_ =0.14 | |
| **Total cholesterol** | | | | | | | | | | |
| < 5.5 mmol/L | 1707 / 110,622 | | | 0.7364 (0.7244, 0.7484) | | | 0.0069 (0.0033, 0.0105) | | 0.0023 (-0.0004, 0.0051) | |
| 5.5 – 6.4 mmol/L | 1859 / 103,583 | | | 0.6947 (0.6822, 0.7071) | | | 0.0164 (0.0111, 0.0217) | | 0.0060 (0.0026, 0.0094) | |
| >=6.4 mmol/L | 2114 / 92,449 | | | 0.6807 (0.6677, 0.6936) | | | 0.0144 (0.0088, 0.0199) | | 0.0048 (0.0020, 0.0076) | |
|  |  | | |  | | | χ_2_=10.3; P_heter_ =0.006 | | χ_2_=3.0; P_heter_ =0.23 | |
| **HDL cholesterol** | | | | | | | | | | |
| < 1.3 mmol/L | 2610 / 98,292 | | | 0.6999 (0.6897, 0.7101) | | | 0.0158 (0.0115, 0.0201) | | 0.0028 (0.0007, 0.0049) | |
| 1.3 – 1.6 mmol/L | 1966 / 114,339 | | | 0.7076 (0.6955, 0.7197) | | | 0.0118 (0.0072, 0.0164) | | 0.0058 (0.0023, 0.0093) | |
| >=1.6 mmol/L | 1104 / 94,023 | | | 0.7079 (0.6899, 0.7259) | | | 0.0069 (0.0010, 0.0129) | | 0.0039 (0.0000, 0.0078) | |
|  |  | | |  | | | χ_1_=5.8; P_heter_ =0.055 | | χ_2_=2.1; P_heter_ =0.34 | |
| **Systolic blood pressure** | | | | | | | | | | |
| < 124 mmHg | 643 / 76,263 | | | 0.7113 (0.6871, 0.7355) | | | 0.0054 (-0.0047, 0.0155) | | 0.0072 (-0.0023, 0.0166) | |
| 124 - 140 mmHg | 1539 / 105,784 | | | 0.6890 (0.6752, 0.7028) | | | 0.0159 (0.0097, 0.0221) | | 0.0067 (0.0027, 0.0106) | |
| >=140 mmHg | 3498 / 124,640 | | | 0.6680 (0.6586, 0.6774) | | | 0.0148 (0.0109, 0.0188) | | 0.0038 (0.0017, 0.0058) | |
|  | | |  | | |  | χ_2_=3.3; P_heter_ =0.19 | | χ_2_=2.0; P_heter_ =0.38 | |
| **10-year CVD risk categories** | | | | | | | | | | |
| < 5% | 3974 / 280,059 | | | 0.6904 (0.6818, 0.6989) | | | 0.0144 (0.0109, 0.0180) | | 0.0053 (0.0031, 0.0075) | |
| 5 - 10% | 1312 / 22,601 | | | 0.5414 (0.5235, 0.5592) | | | 0.0413 (0.0264, 0.0562) | | 0.0224 (0.0105, 0.0343) | |
| >=10% | 394 / 3994 | | | 0.5257 (0.4927, 0.5588) | | | 0.0283 (0.0042, 0.0525) | | 0.0076 (-0.0054, 0.0206) | |
|  | | |  | | |  | χ_2_=12.8; P_heter_ =0.002 | | χ_2_=7.7; P_heter_ =0.021 | |

Conventional risk factors included information on age at baseline, sex, smoking status, history of diabetes, systolic blood pressure, total cholesterol and HDL-cholesterol. Prediction model was developed using Cox regression for all participants, stratified by study centre and sex, adjusted for conventional risk predictors, where appropriate. Polygenic risk scores included the polygenic risk score for CHD, and the one for ischaemic stroke (see **Fig 2**) as two linear predictors in the model throughout.
