# Supplementary material for: Polygenic risk scores in cardiovascular risk prediction: A cohort study and modelling analyses
Source: PLoS Med. 2021 Jan 14;18(1):e1003498. doi: 10.1371/journal.pmed.1003498 (PMC7808664; doi:10.1371/journal.pmed.1003498)
Supplement: S4 Table — Conventional risk factors included age at baseline, sex, smoking status, history of diabetes, systolic blood pressure, total cholesterol, and HDL cholesterol, with stratification by study centre and sex, where appropriate. (DOCX) [file pmed.1003498.s018.docx]

| **S4 Table. Partial likelihood ratio test for models with polygenic risk scores beyond conventional risk factors, C-reactive protein, and treatment of hypertension** | | |
| --- | --- | --- |
|  | **Partial likelihood-ratio test (χ^2^)** | **C-index changes**  **(95% CI)** |
| Conventional risk factors | Reference | |
| Conventional risk factors, plus PRSs | 434 | 0.012 (0.009, 0.015) |
|  |  |  |
| Conventional risk factors, plus C-reactive protein | Reference | |
| Conventional risk factors, plus C-reactive protein, and PRSs | 428 | 0.012 (0.009, 0.014) |
|  |  |  |
| Conventional risk factors, plus treatment of hypertension | Reference | |
| Conventional risk factors, plus treatment of hypertension, and PRSs | 420 | 0.012 (0.009, 0.014) |

Conventional risk factors included information age at baseline, sex, smoking status, history of diabetes, systolic blood pressure, total cholesterol, HDL-cholesterol, with stratification of study centre and sex, where appropriate.
