# Supplementary material for: Polygenic risk scores in cardiovascular risk prediction: A cohort study and modelling analyses
Source: PLoS Med. 2021 Jan 14;18(1):e1003498. doi: 10.1371/journal.pmed.1003498 (PMC7808664; doi:10.1371/journal.pmed.1003498)
Supplement: S5 Table — ACC, American College of Cardiology; AHA, American Heart Association; NICE, National Institute for Health and Care Excellence. *Conventional risk factors included age, sex, smoking, systolic blood pressure, history of diabetes, total cholesterol, and HDL cholesterol. Polygenic risk scores included the polygenic risk score for CHD and the one for ischaemic stroke (see Fig 2) as 2 linear predictors in the model throughout. Calculations of the above categorical NRIs were <5%, 5% to <7.5%, and ≥7.5% according to the 2019 ACC/AHA guideline, and <5%, 5% to <10%, and ≥10% according to the 2014 NICE guideline. (DOCX) [file pmed.1003498.s019.docx]

| **S5 Table. Net reclassification index (NRI) for incident cardiovascular disease by addition of information on polygenic risk scores, and C-reactive protein, above conventional risk factors, for non-cases, and cases, including participants on statin treatment at baseline** | | | | | | | | |
| --- | --- | --- | --- | --- | --- | --- | --- | --- |
| **Conventional risk factors** | | **Categorical NRI (%, 95% CI)**  **vs Reference model** | | | | **Continuous NRI (%, 95% CI)** | | |
|  |  | **Before recalibration** | | **After recalibration** | | **Overall** | **Men** | **Women** |
|  | | **NICE 2014** | **AHA/ACC 2019** | **NICE 2014** | **AHA/ACC 2019** |  |  |  |
| **Plus polygenic risk scores alone** | | |  |  |  |  |  |  |
| Non-cases |  | -0.5 (-0.7, -0.3) | -0.6 (-0.8, -0.3) | 1.8 (1.4, 2.1) | 2.4 (2.1, 2.8) | 12.0 (11.7, 12.4) | 13.3 (12.8, 13.8) | 9.7 (9.2, 10.1) |
| Cases |  | 3.9 (3.1, 4.7) | 4.6 (3.7, 5.4) | 1.1 (0.2, 2.0) | 0.3 (-0.6, 1.1) | 9.7 (7.3, 12.2) | 10.6 (7.5, 13.7) | 7.5 (2.9, 12.2) |
| **Plus C-reactive protein alone** | | |  |  |  |  |  |  |
| Non-cases |  | -0.3 (-0.5, -0.2) | -0.3 (-0.5, -0.2) | 0.5 (0.2, 0.8) | 0.7 (0.4, 1.1) | 12.3 (11.9, 12.7) | 13.1 (12.6, 13.6) | 10.4 (9.9, 10.8) |
| Cases |  | 1.9 (1.3, 2.5) | 2.3 (1.6, 3.0) | 0.6 (-0.1, 1.3) | 0.1 (-0.6. 0.8) | 2.5 (-0.3, 5.3) | 2.3 (-1.3, 5.8) | 3.8 (-1.0, 8.6) |
| ACC=American College of Cardiology; AHA=American Heart Association; NICE=National Institute for Health and Care Excellence; NRI=Net reclassification improvement; *Conventional risk factors included information on age, sex, smoking, systolic blood pressure, history of diabetes, total cholesterol, and HDL cholesterol. Polygenic risk scores included the polygenic risk score for CHD, and the one for ischaemic stroke (see **Fig 2**) as two linear predictors in the model throughout. Calculations of the above categorical NRIs were: <5%, 5-7.5%, and ≥7.5% according to the 2019 ACC/AHA guideline, and <5%, 5-10%, ≥10% according to the 2014 NICE guideline. | | | | | | | | |
