# Supplementary material for: Polygenic risk scores in cardiovascular risk prediction: A cohort study and modelling analyses
Source: PLoS Med. 2021 Jan 14;18(1):e1003498. doi: 10.1371/journal.pmed.1003498 (PMC7808664; doi:10.1371/journal.pmed.1003498)
Supplement: S6 Table — CVD, cardiovascular disease; CHD, coronary heart disease; IS, ischaemic stroke; CI, confidence interval. Conventional risk factors included age at baseline, sex, smoking status, history of diabetes, systolic blood pressure, total cholesterol, and HDL cholesterol, with stratification by study centre and sex, where appropriate. The PRS for CHD and the PRS for IS were constructed using methods as in our previous work [1]. The PRS for stroke was constructed using the genome-wide significant variants in the MEGASTROKE consortium for total stroke, and linkage-disequilibrium-thinned in UK Biobank, with corresponding weights taken from the MEGASTROKE consortium [2]. Construction procedures of the 2 above PRSs did not include estimates from previous GWASs on other vascular risk factors. The PRS for IS was constructed using methods described in our previous work [3], by taking account of 19 phenotypes, and is publicly available (https://www.pgscatalog.org/score/PGS000039/). The PRS for CVD was constructed using the same approach as the PRS for IS but with CVD as the outcome. (DOCX) [file pmed.1003498.s020.docx]

| **S6 Table. Comparison of different polygenic risk scores (PRSs) on strength of associations, discriminative ability, and reclassification index for different cardiovascular outcomes, in UK Biobank** | | | | |
| --- | --- | --- | --- | --- |
|  | **CVD** | **CHD** | **Stroke** | **Ischaemic stroke** |
|  | **n=5680** | **n=3333** | **n=2347** | **n=1477** |
| **Age- and sex-adjusted hazards ratios (95% CI)** | |  |  |  |
| PRS for CHD | 1.36 (1.32, 1.40) | 1.56 (1.51, 1.62) | 1.11 (1.07, 1.16) | 1.17 (1.11, 1.23) |
| PRS for IS | 1.22 (1.19, 1.26) | 1.25 (1.21, 1.29) | 1.19 (1.14, 1.24) | 1.21 (1.15, 1.28) |
| PRS for stroke | 1.13 (1.10, 1.16) | 1.14 (1.10, 1.18) | 1.12 (1.08, 1.17) | 1.11 (1.06, 1.17) |
| PRS for CVD | 1.38 (1.34, 1.41) | 1.54 (1.49, 1.60) | 1.17 (1.12, 1.22) | 1.21 (1.15, 1.28) |
| **C-index changes (95% CI) vs. conventional risk factors** | | | | |
| PRS for CHD | 0.0107 (0.0081, 0.0132) | 0.0211 (0.0167, 0.0255) | 0.0012 (0.0000, 0.0025) | 0.0029 (0.0008, 0.0051) |
| PRS for IS | 0.0044 (0.0028, 0.0060) | 0.0048 (0.0027, 0.0069) | 0.0031 (0.0010, 0.0052) | 0.0045 (0.0018, 0.0072) |
| PRS for stroke | 0.0016 (0.0006, 0.0025) | 0.0019 (0.0006, 0.0032) | 0.0008 (0.0004, 0.0012) | 0.0009 (0.0005, 0.0013) |
| PRS for CVD | 0.0105 (0.0081, 0.0129) | 0.0180 (0.0140, 0.0221) | 0.0024 (0.0006, 0.0041) | 0.0038 (0.0013, 0.0062) |
|  |  |  |  |  |
| PRS for CHD + PRS for IS | 0.0119 (0.0093, 0.0145) | 0.0216 (0.0172, 0.0261) | 0.0035 (0.0013, 0.0056) | 0.0058 (0.0028, 0.0087) |
| PRS for CHD + PRS for stroke | 0.0115 (0.0089, 0.0140) | 0.0219 (0.0174, 0.0263) | 0.0019 (0.0002, 0.0036) | 0.0036 (0.0012, 0.0059) |
| **Continuous net reclassification index (95% CI)** | | | | |
| PRS for CHD | 0.2068 (0.1785, 0.2351) | 0.3157 (0.2778, 0.3536) | 0.0695 (0.0230, 0.1159) | 0.1329 (0.0709, 0.1949) |
| PRS for IS | 0.1202 (0.0896, 0.1508) | 0.1409 (0.1024, 0.1794) | 0.0848 (0.0386, 0.1309) | 0.1061 (0.0508, 0.1614) |
| PRS for stroke | 0.0654 (0.0368, 0.0939) | 0.1005 (0.0536, 0.1474) | 0.0559 (0.0101, 0.1018) | 0.0455 (-0.0144, 0.1055) |
| PRS for CVD | 0.2222 (0.1948, 0.2496) | 0.2970 (0.2612, 0.3328) | 0.1170 (0.0689, 0.1652) | 0.1612 (0.1036, 0.2188) |
|  |  |  |  |  |
| PRS for CHD + PRS for IS | 0.2274 (0.1970, 0.2578) | 0.3209 (0.2817, 0.3602) | 0.1005 (0.0536, 0.1474) | 0.1591 (0.0968, 0.2213) |
| PRS for CHD + PRS for stroke | 0.2233 (0.1941, 0.2525) | 0.3259 (0.2895, 0.3623) | 0.0885 (0.0431, 0.1340) | 0.1425 (0.0870, 0.1979) |

CVD, cardiovascular disease; CHD, coronary heart disease; IS, ischaemic stroke; CI, confidence intervals; Conventional risk factors included information age at baseline, sex, smoking status, history of diabetes, systolic blood pressure, total cholesterol, HDL-cholesterol, with stratification of study centre and sex, where appropriate. PRS for CHD, and PRS for IS were constructed using methods as in our previous work [1]. PRS for stroke was constructed using the genome-wide significant variants in MEGASTROKE consortium for total stroke, and LD thinned in UKB, with corresponding weights taken from the MEGASTROKE consortium [2]. Construction procedures of the two above PRSs did not include estimates from previous GWAS studies on other vascular risk factors. PRS for IS was constructed using methods described in our previous work [3], by taking account of 19 phenotypes, and publically available (<https://www.pgscatalog.org/score/PGS000039/>). PRS for CVD was constructed using the same approach as PRS for IS with CVD as the outcome.

**References:**

1. Inouye M, Abraham G, Nelson CP, Wood AM, Sweeting MJ, Dudbridge F, et al. Genomic Risk Prediction of Coronary Artery Disease in 480,000 Adults: Implications for Primary Prevention. JACC. 2018;72(16):1883-93. doi: http://doi.org/10.1016/j.jacc.2018.07.079. PubMed PMID: 30309464

2. Malik R, Chauhan G, Traylor M, Sargurupremraj M, Okada Y, Mishra A, et al. Multiancestry genome-wide association study of 520,000 subjects identifies 32 loci associated with stroke and stroke subtypes. Nat Genet. 2018;50(4):524-37. doi: http://doi.org/10.1038/s41588-018-0058-3. PubMed PMID: 29531354.

3. Torkamani A, Wineinger NE, Topol EJ. The personal and clinical utility of polygenic risk scores. Nat Rev Genet. 2018;19(9):581-90. doi: https://doi.org/10.1038/s41576-018-0018-x. PubMed PMID: 29789686.
