# Supplementary material for: Polygenic risk scores in cardiovascular risk prediction: A cohort study and modelling analyses
Source: PLoS Med. 2021 Jan 14;18(1):e1003498. doi: 10.1371/journal.pmed.1003498 (PMC7808664; doi:10.1371/journal.pmed.1003498)
Supplement: S7 Table — *The PCE score for study participants in UK Biobank was calculated using the updated Pooled Cohort Equations score, i.e., the weights for each constituent predictor variable, as previously published [1]. **PCE variables included age at baseline, sex, smoking status, history of diabetes, systolic blood pressure, total cholesterol, HDL cholesterol, ethnicity, and treatment of high blood pressure, weighted by the Cox regression coefficients estimated in UK Biobank. Polygenic risk scores included the polygenic risk score for CHD and the one for ischaemic stroke (see Fig 2) as 2 linear predictors in the model throughout. (DOCX) [file pmed.1003498.s021.docx]

| **S7 Table. Incremental predictive ability of polygenic risk scores, and C-reactive protein, above the updated Pooled Cohort Equations score (PCE)** | | | |
| --- | --- | --- | --- |
|  | **C-index**  **(95% CI)** | **C-index changes**  **(95% CI)** |  |
| PCE 2013 (i.e., the published algorithm)* | 0.708 (0.701, 0.715) | Reference |  |
| PCE, plus C-reactive protein | 0.713 (0.706, 0.720) | 0.005 (0.004, 0.007) |  |
| PCE, plus PRSs | 0.721 (0.714, 0.728) | 0.013 (0.011, 0.016) |  |
| PCE, plus C-reactive protein, and PRSs | 0.725 (0.718, 0.731) | 0.017 (0.015, 0.020) |  |
|  |  |  |  |
| PCE variables** | 0.712 (0.705, 0.719) | Reference |  |
| PCE variables, plus C-reactive protein | 0.715 (0.709, 0.722) | 0.004 (0.002, 0.005) |  |
| PCE variables, plus PRSs | 0.723 (0.716, 0.730) | 0.012 (0.009, 0.014) |  |
| PCE variables, plus C-reactive protein, and PRSs | 0.727 (0.720, 0.733) | 0.015 (0.012, 0.018) |  |
| *The PCE score for study participants in UK Biobank was calculated using the updated pooled cohort equations score, i.e., the weights for each constituent predictor variable, as previously published [1].**PCE variables included age at baseline, sex, smoking status, history of diabetes, systolic blood pressure, total cholesterol, HDL-cholesterol, ethnicity, and treatment of high blood pressure, weighted by the Cox regression coefficients estimated in UK Biobank. Polygenic risk scores included the polygenic risk score for CHD, and the one for ischaemic stroke (see **Fig 2**) as two linear predictors in the model throughout. | | | |

**Reference:**

1. Stone Neil J, Robinson Jennifer G, Lichtenstein Alice H, Bairey Merz CN, Blum Conrad B, Eckel Robert H, et al. 2013 ACC/AHA Guideline on the Treatment of Blood Cholesterol to Reduce Atherosclerotic Cardiovascular Risk in Adults. Circulation. 2014;129(25_suppl_2):S1-S45. doi: http://doi.org/10.1161/01.cir.0000437738.63853.7a. PubMed PMID: 24222016.
