# Supplementary material for: Polygenic risk scores in cardiovascular risk prediction: A cohort study and modelling analyses
Source: PLoS Med. 2021 Jan 14;18(1):e1003498. doi: 10.1371/journal.pmed.1003498 (PMC7808664; doi:10.1371/journal.pmed.1003498)
Supplement: S8 Table — Conventional risk factors included age at baseline, sex, smoking status, history of diabetes, systolic blood pressure, total cholesterol, and HDL cholesterol. Polygenic risk scores included the polygenic risk score for CHD and the one for ischaemic stroke (see Fig 2) as 2 linear predictors in the model throughout. Cumulative incidence of the composite CVD outcomes was estimated using the cause-specific hazards ratios from Cox regression, in the presence of competing risk from non-CVD deaths. (DOCX) [file pmed.1003498.s022.docx]

| **S8 Table: Incremental predictive ability of polygenic risk scores, and C-reactive protein, with or without adjusting for competing risk from non-cardiovascular death** | | | | | |
| --- | --- | --- | --- | --- | --- |
|  | **Without adjustment for competing risk** | |  | **With adjustment for competing risk** | |
|  | **Overall C-index**  **(95% CI)** | **C-index changes**  **(95% CI)** |  | **Overall C-index**  **(95% CI)** | **C-index changes**  **(95% CI)** |
| Conventional risk factors | 0.710 (0.703, 0.717) | Reference |  | 0.709 (0.702, 0.715) | Reference |
| Plus C-reactive protein alone | 0.714 (0.707, 0.721) | 0.004 (0.003, 0.006) |  | 0.714 (0.708, 0.721) | 0.006 (0.004, 0.008) |
| Plus PRSs alone | 0.722 (0.716, 0.730) | 0.012 (0.009, 0.015) |  | 0.722 (0.716, 0.729) | 0.014 (0.011,0.016) |
| Plus the above both | 0.725 (0.719, 0.732) | 0.016 (0.017, 0.019) |  | 0.726 (0.719, 0.732) | 0.017 (0.014,0.020) |
| Conventional risk factors included age at baseline, sex, smoking status, history of diabetes, systolic blood pressure, total cholesterol, and HDL-cholesterol. Polygenic risk scores included the polygenic risk score for CHD, and the one for ischaemic stroke (see **Fig 2**) as two linear predictors in the model throughout. Cumulative incidence of the composite CVD outcomes were estimated using the cause-specific hazards ratios from Cox regression, in the presence of competing risk from non-CVD deaths. | | | | | |
