# Supplementary material for: Polygenic risk scores in cardiovascular risk prediction: A cohort study and modelling analyses
Source: PLoS Med. 2021 Jan 14;18(1):e1003498. doi: 10.1371/journal.pmed.1003498 (PMC7808664; doi:10.1371/journal.pmed.1003498)
Supplement: S9 Table — PRS, polygenic risk score; CRP, C-reactive protein. Conventional risk factors included age at baseline, sex, smoking, systolic blood pressure, history of diabetes, total cholesterol, and HDL cholesterol. Polygenic risk scores included the polygenic risk score for CHD and the one for ischaemic stroke (see Fig 2) as 2 linear predictors in the model throughout. The predicted 10-year cardiovascular risk categories used the 2014 NICE guideline. Estimates of public health impact for a hypothetical population of 100,000 individuals (40–75 years) were based on (1) the sex- and age-specific (5-year) profile of a standard UK population (2017 mid-year population) [35] and (2) sex-specific 5-year age-at-risk incidence rates of cardiovascular disease in the CPRD, among individuals without prior history of cardiovascular disease and not on statin treatment at baseline. Estimates for public health impact are shown before and after recalibration. (DOCX) [file pmed.1003498.s023.docx]

| **S9 Table. Estimates of public health impact with targeted assessment (intermediate-risk: 5-10%) of polygenic risk scores, and C-reactive protein among 100,000 UK adults** | | | | | | |
| --- | --- | --- | --- | --- | --- | --- |
|  | **Before recalibration** | |  | | **After recalibration** | |
|  | **0-10%** | **5-10%** |  |  | **0-10%** | **5-10%** |
| ***Additional cases identified in addition to conventional risk factors (%)*** | | | | | | |
| Plus PRSs only | 15.9 | 15.9 |  | 7.1 | | 7.1 |
| Plus CRP only | 9.2 | 9.2 |  | 4.8 | | 4.8 |
| ***Number needed to screen per event prevented in addition to conventional risk factors*** | | | | | | |
| *Number to screen* | *89,387* | *8871* |  | *68,836* | | *23,972* |
| Plus PRSs only | 1781 | 178 |  | 961 | | 336 |
| Plus CRP only | 3020 | 300 |  | 1411 | | 491 |
| PRSs, Polygenic risk scores; CRP, C-reactive protein; Conventional risk factors included information on age at baseline, sex, smoking, systolic blood pressure, history of diabetes, total cholesterol and HDL-cholesterol. Polygenic risk scores included the polygenic risk score for CHD, and the one for ischaemic stroke (see **Fig 2**) as two linear predictors in the model throughout. The predicted 10-year cardiovascular risk categories used 2014 NICE guideline. Estimates of public health impact for a hypothetical population of 100,000 individuals (40-75 years) were based on: 1) sex- and age-specific (5-year) profile of a standard UK population (2017 mid-year population, <https://www.ons.gov.uk/>); 2) sex-specific 5-year age-at-risk incidence rates of cardiovascular disease in CPRD, among individuals without prior history of cardiovascular disease, and not on statin treatment at baseline; 3) estimates for public health impact were shown, respectively, before and after recalibration. | | | | | | |
