# Supplementary material for: Polygenic risk scores in cardiovascular risk prediction: A cohort study and modelling analyses
Source: PLoS Med. 2021 Jan 14;18(1):e1003498. doi: 10.1371/journal.pmed.1003498 (PMC7808664; doi:10.1371/journal.pmed.1003498)
Supplement: S11 Table — *Among cases and non-cases, respectively, 1,197 and 7,354 participants had diabetes or LDL cholesterol measurement of 5.0 mmol/l or greater. Numbers in red are shown for individuals who were reclassified downwards with additional assessment, and numbers in blue are shown for individuals who were reclassified upwards with additional assessment. Polygenic risk scores included the polygenic risk score for CHD and the one for ischaemic stroke (see Fig 2) as 2 linear predictors in the model throughout. (DOCX) [file pmed.1003498.s025.docx]

**S11 Table. Numerical results for estimates of public health impact by additional assessment of polygenic risk scores (PRSs) or C-reactive protein, above conventional risk predictors, among 100,000 individuals**

| **Conventional risk factors** | **Plus PRSs alone** | | |  | **Plus C-reactive protein alone** | | |
| --- | --- | --- | --- | --- | --- | --- | --- |
|  | **0-<5%** | **5-<10%** | **≥10%** |  | **0-<5%** | **5-<10%** | **≥10%** |
| **Cases (n=7997)*** | | | | | | | |
| 0-<5% | 899 | 182 | 1 |  | 977 | 105 | 0 |
| 5-<10% | 198 | 1307 | 357 |  | 90 | 1528 | 244 |
| ≥10% | 0 | 271 | 3586 |  | 0 | 180 | 3677 |
|  | | | | | | | |
| **Non-cases (n=92,003)*** | | | | | | | |
| 0-<5% | 40,939 | 2833 | 12 |  | 41,911 | 1871 | 0 |
| 5-<10% | 4086 | 15,267 | 2758 |  | 2238 | 18,149 | 1723 |
| ≥10% | 24 | 3110 | 15,619 |  | 0 | 1526 | 17,226 |

*Among cases and non-cases, respectively, 1197 and 7354 participants had diabetes or LDL cholesterol measurement of 5.0 mmol/L or greater. Numbers in red are shown for individuals who were reclassified downwards with additional assessment, and numbers in blue are shown for individuals who were reclassified upwards with additional assessment. Polygenic risk scores included the polygenic risk score for CHD, and the one for ischaemic stroke (see **Fig 2**) as two linear predictors in the model throughout.
