# Supplementary material for: Polygenic risk scores in cardiovascular risk prediction: A cohort study and modelling analyses
Source: PLoS Med. 2021 Jan 14;18(1):e1003498. doi: 10.1371/journal.pmed.1003498 (PMC7808664; doi:10.1371/journal.pmed.1003498)
Supplement: S1 Text — (DOCX) [file pmed.1003498.s026.docx]

**S1 Text. Description of analytic dataset from UK Biobank**

Details of UK Biobank (UKB) have been described previously [1]. Briefly, over 500,000 participants aged 40-69 years were recruited during 2006-2010 in 22 geographical centres throughout the United Kingdom, covering a variety of different settings to provide socioeconomic and ethnic heterogeneity and an urban-rural mix. The assessment visit comprised electronic signed consent; a self-completed touch-screen questionnaire; brief computer-assisted interview; physical and functional measures; and collection of biological samples for all participants at recruitment for long-term storage. All participants were followed-up through linkages to routinely available national datasets, including Hospital Episode Statistics (HES) data, primary care, cancer screening data, and disease-specific registries [2]. The UK Biobank study was approved by the North West Multi-centre Research Ethics Committee, and all participants provided written informed consent to participate in the UKB.

Genotyping was undertaken using a custom-built genome-wide array of ~826,000 markers. Imputation to ∼96 million markers was subsequently carried out using the Haplotype Reference Consortium and UK10K/1000Genomes reference panels. All genetic analyses utilised the UKB phase 3 release imputed genotype data [3]. Thirty circulating clinical biochemistry markers were measured in serum or red blood cells. These markers were selected for measurement in UKB for various reasons, including their reflection of established risk factors for chronic diseases, established use as diagnostic measures, and/or ability to reflect phenotypes not otherwise well-assessed or feasibly measured at scale [4, 5].

HES data available for the current analysis covered hospital admissions to NHS hospitals in England from 1996 to 2017, with the Scottish data dating back as early as 1981. HES used International Classification of Diseases (ICD)–9th and 10th Revisions to record diagnosis information, and Office of Population, Censuses and Surveys: Classification of Interventions and Procedures, version 4 (OPCS-4) to code operative procedures. Death registries provided data on deaths in the UK until 2017, with both primary and contributory causes of death coded in ICD-10. Information on smoking status (current-smoker vs. others) was obtained using the touchscreen questionnaire at baseline visit (Data-Field 20116). History of diabetes at baseline (yes vs. no) was defined using self-reported information recorded at baseline, and information collected at resurveys to populate or correct baseline data, where appropriate (Data-Fields: 2443, 4041, 10844, 20002, and 20008). Systolic blood pressure (mmHg) was measured twice on the left arm while participants were seated, using an Omron blood pressure monitor, and the mean value of the two measurements was used in the present analyses. Prior history of vascular disease was defined using self-reported information recorded at the baseline visit in UKB and updated using information on hospitalization before baseline extracted from HES. Prior vascular diseases included coronary heart disease, other heart disease, stroke, transient ischaemic stroke, peripheral vascular disease, angina, or cardiovascular surgery. Lipid-lowering status (current vs. others) was obtained via self-reported information at baseline (Data-Fields: 6177 for men and 6153 for women, respectively).

**References**

1. Sudlow C, Gallacher J, Allen N, Beral V, Burton P, Danesh J, et al. UK biobank: an open access resource for identifying the causes of a wide range of complex diseases of middle and old age. PLoS Med. 2015;12(3):e1001779. <http://doi.org/10.1371/journal.pmed.1001779>. PMID: 25826379.

2. UK Biobank. Protocol for a large-scale prospective epidemiological resource (2007) [01/July/2019]. Available from: <http://www.ukbiobank.ac.uk/wp-content/uploads/2011/11/UK-Biobank-Protocol.pdf>.

3. Bycroft C, Freeman C, Petkova D, Band G, Elliott LT, Sharp K, et al. Genome-wide genetic data on ~500,000 UK Biobank participants. bioRxiv. 2017:166298. 10.1101/166298.

4. Fry D, Almond R, Moffat S, Gordon M, Singh P. UK Biobank Biomarker Project: Companion Document to Accompany Serum Biomarker Data (2019). <http://biobank.ctsu.ox.ac.uk/showcase/docs.cgi?id=0>: Contract No.: 22 Apr, 2019.

5. Arnold M, Parish S. Biomarker assay quality procedures: approaches used to minimise systematic and random errors (and the wider epidemiological implications) (2019) [22 Apr, 2019]. Available from: <http://biobank.ctsu.ox.ac.uk/showcase/docs/biomarker_issues.pdf>.
