# Supplementary material for: Polygenic risk scores in cardiovascular risk prediction: A cohort study and modelling analyses
Source: PLoS Med. 2021 Jan 14;18(1):e1003498. doi: 10.1371/journal.pmed.1003498 (PMC7808664; doi:10.1371/journal.pmed.1003498)
Supplement: S2 Text — (DOCX) [file pmed.1003498.s027.docx]

**S2 Text. Description of analytic dataset from Clinical Practice Research Datalink**

The Clinical Practice Research Datalink (CPRD) is an ongoing primary care database of anonymised medical records from general practitioners, with coverage of over 11.3 million patients from 674 practices in the UK (England, Wales, Scotland, and Northern Ireland), with data collected from 1987 onwards. Patients are broadly representative of the UK general population in terms of age, sex and ethnicity [1]. A subset of English practices (currently 75%, representing 58% of all UK CPRD practices) have consented to participate in the CPRD linkage scheme and have provided patient-level information. Patient-level data from consenting practices are linked via a trusted third party to other existing data sources, including HES and Office for National Statistics (ONS). Records on about 2.1 million patients were used in the present analysis (**Fig A**).

**Disease and exposure definitions**

CVD was defined by ICD-10 codes: I21-I25 (fatal and non-fatal events), and I60-69 (fatal and non-fatal). Non-fatal hospital inpatient admissions using these codes were flagged in HES. Fatal events were flagged using death certificate information (coded using ICD-10) in the ONS data. The primary care database in CPRD is coded using medcodes (a CPRD-unique classification system), which are directly mapped to Read version 3 (CTV3) codes. Mapping of ICD-10 CVD codes to CTV3 codes was done using the NHS Digital TRUD Data Migration Toolkit [2]; the complete list of CTV3 codes generated is available on request. An individual’s date of first CVD event was calculated as the earliest event flagged in any of the three sources of information. Current smokers were flagged using a code list previously reported [3]. Diabetes diagnoses were flagged by: prescriptions for anti-diabetic medications [4]; CTV3 codes indicating a diabetes diagnosis [5]; or ICD-10 codes in HES (E10-E14, G59.0, G63.2, H28.0, H36.0, Y42.3). Statin prescriptions were flagged according to a list of statin medications [3]. Full details of the code lists used are available on request.

**Calculation of CVD incidence rates**

An individual’s date of entry into the CPRD cohort was defined as the latest of: 6 months after registration at GP practice contributing to CPRD; date of 30^th^ birthday; date GP practice data quality marked as “up to standard”; 1^st^ April 2004. The date of cohort exit was defined as the earliest of: date of de-registering at practice; date of last data upload from practice to CPRD; date of 95^th^ birthday; date of death; or 21^st^ Oct, 2017. Participants were assumed to be at-risk for CVD from study entry until first CVD event or censoring. The age- and sex-specific incidence rates of CVD were calculated among individuals with no record of having either CVD or a statin prescription prior to the date of study entry (**Table A in S2 Text**).

| **Table A in S2 Text: Age- and sex-specific incidence rates (per 1000 person-years) of CVD among CPRD participants (n=2,156,627)** | | | |
| --- | --- | --- | --- |
| **Age-at-risk** |  | **Male** | **Female** |
| 40-<45 |  | 1.578 | 0.726 |
| 45-<50 |  | 2.964 | 1.309 |
| 50-<55 |  | 5.141 | 2.165 |
| 55-<60 |  | 7.561 | 2.969 |
| 60-<65 |  | 10.848 | 4.560 |
| 65-<70 |  | 13.731 | 7.007 |
| 70-<75 |  | 19.271 | 10.840 |
| 75-<80 |  | 25.667 | 17.007 |

**Fig A in S2 Text: Study population in the Clinical Practice Research Datalink**

**
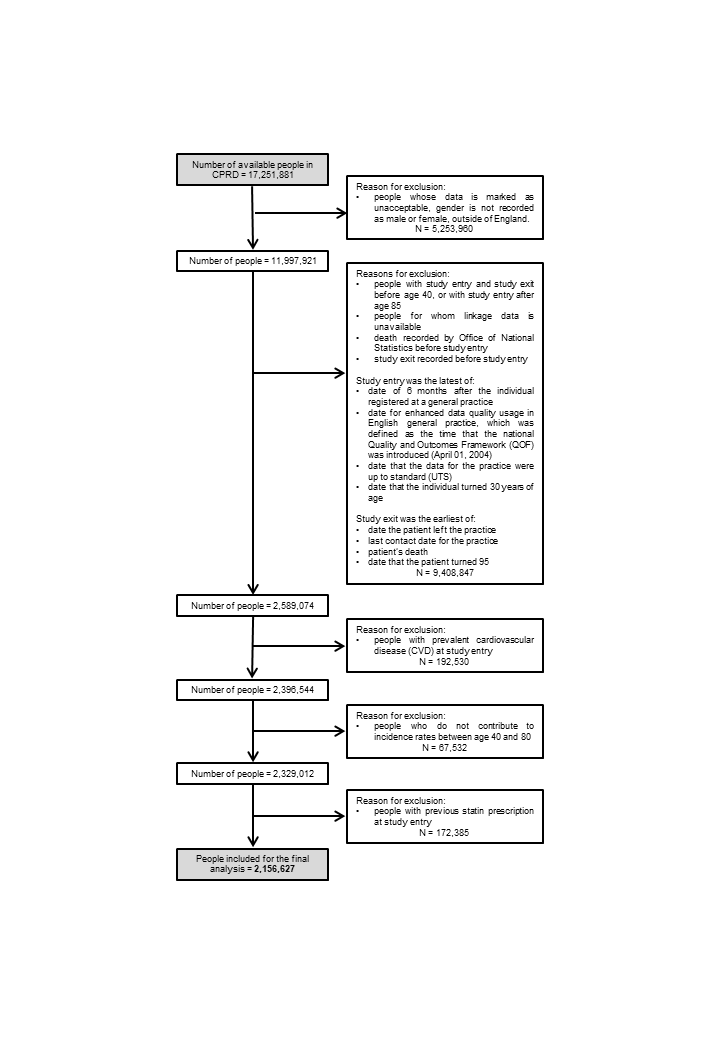
**

**References**

1. Herrett E, Gallagher AM, Bhaskaran K, Forbes H, Mathur R, van Staa T, et al. Data Resource Profile: Clinical Practice Research Datalink (CPRD). Int J Epidemiol. 2015;44(3):827-36. Epub 2015/06/08. <http://doi.org/10.1093/ije/dyv098>. PMID: 26050254.

2. National Health Service (NHS). NHS Digital TRUD (2019) <https://isd.digital.nhs.uk/trud3/user/guest/group/0/pack/9/subpack/9/releases> [26 June, 2019]. Available from: <https://isd.digital.nhs.uk/trud3/user/guest/group/0/pack/9/subpack/9/releases>.

3. Reeves D, Springate DA, Ashcroft DM, Ryan R, Doran T, Morris R, et al. Can analyses of electronic patient records be independently and externally validated? The effect of statins on the mortality of patients with ischaemic heart disease: a cohort study with nested case–control analysis. BMJ Open. 2014;4(4):e004952. <http://doi.org/10.1136/bmjopen-2014-004952>. PMID: 24760353

4. Kontopantelis E, Springate DA, Reeves D, Ashcroft DM, Rutter MK, Buchan I, et al. Glucose, blood pressure and cholesterol levels and their relationships to clinical outcomes in type 2 diabetes: a retrospective cohort study. Diabetologia. 2015;58(3):505-18. Epub 2014/12/17. <http://doi.org/10.1007/s00125-014-3473-8>. PMID: 25512005.

5. Wright AK, Kontopantelis E, Emsley R, Buchan I, Sattar N, Rutter MK, et al. Life Expectancy and Cause-Specific Mortality in Type 2 Diabetes: A Population-Based Cohort Study Quantifying Relationships in Ethnic Subgroups. Diabetes care. 2017;40(3):338-45. Epub 2016/12/22. <http://doi.org/10.2337/dc16-1616>. PMID: 27998911.
