# Supplementary material for: Polygenic risk scores in cardiovascular risk prediction: A cohort study and modelling analyses
Source: PLoS Med. 2021 Jan 14;18(1):e1003498. doi: 10.1371/journal.pmed.1003498 (PMC7808664; doi:10.1371/journal.pmed.1003498)
Supplement: S3 Text — (DOCX) [file pmed.1003498.s028.docx]

**S3 Text. Statistical methods used for estimating public health impact**

The general process taken to estimate the potential public health impact of using different risk models for population screening is provided in **Fig A in S3 Text**, and broadly involved three pieces of information.

1. Predicted 9-year CVD risk in UKB participants using models including conventional risk predictors, PRS and the nine selected clinical biochemistry markers.
2. Incidence rates of CVD by sex and 5-year age-at-risk among individuals without prior history of CVD, and not on statin treatment at baseline in CPRD.
3. UK population structure by sex and 5-year age groups in mid-2017, from UK office of national statistics.

**Calculation of 10-year CVD risk for UK Biobank participants**

Since the maximum follow-up of participants in majority of UKB centres was less than 10 years (median follow-up was 8.1 years), we opted to calculate the 9-year CVD risk for each participant as an approximation to their 10-year CVD risk, using models including the different risk predictors (e.g., conventional risk predictors, PRS, and clinical biochemistry markers). Proportional hazards assumption by centre was not violated.

**Recalibration of 10-year CVD risk for UK Biobank participants**

Given that UKB participants have been found to be, on average, healthier than the UK general population (**Fig B in S3 Text**), absolute risk estimates derived from UKB participants are lower than those estimated by deriving and applying risk models in a general population. This can be attributed to the higher baseline survival probability, generally lower values of risk factors, and shorter follow-up period in UKB. Crude reclassification statistics relying on clinically relevant risk thresholds, calculated within the UKB dataset are, therefore, not generalizable to a broader UK primary prevention setting. To correct for this, we adapted (i.e., recalibrated) the predicted 9-year CVD risk for each UKB participant, using incidence rates estimated in CPRD. The general recalibration process has been previously described [11], and involves a simple rescaling of the participants’ risk predictions without affecting the ability of the model to discriminate risk. For the current analysis, recalibration was undertaken separately for each model, using the following steps:

1. In UKB, we estimated the 9-year CVD risk $({\hat{\text{risk}}}_{\text{pred},i}\left( 9 \right)$) for individual $i$ using a Cox model including the relevant set of risk predictors.
2. In UKB, we calculated the mean of the predicted 9-year CVD risks for each sex and 5-year age group (${\hat{\text{risk}}}_{\text{pred},\text{agegrp}}\left( 9 \right)$).
3. In CPRD, among individuals without prior history of CVD, and not on statin treatment at registration, we calculated the incidence rates of CVD for each sex and 5-year age-at-risk group. Assuming exponential survival (i.e., constant hazard) within each 5-year age group, the expected 10-year CVD risk was estimated as follows:

${\hat{\text{risk}}}_{\text{expected}}\left( 10 \right)=1 - exp(-{IR}_{\text{mid}}\times10)$ (1)

where ${IR}_{\text{mid}}$ is the annual incidence at the mid-point of the 10 year interval ahead, i.e., for the 40 to 44 year age-group the incidence rate for 45 to 49 years was used.

1. The following recalibration model was fitted relating the expected risk to the means of predicted risks by age group, for each sex, with transformation applied.

$g\left( 1-{\hat{\text{risk}}}_{\text{expected}}\left( 10 \right) \right)=\alpha+ \beta g\left( 1-{\hat{\text{risk}}}_{\text{pred}}(9) \right)$ (2)

where $g$(.) is the link function $ln(-ln(.)$)

1. $\hat{\alpha}$and$\hat{\beta}$ from the fitted recalibration model are then used to adjust the original 9-year risk prediction ${\hat{\text{risk}}}_{\text{pred},i}\left( 9 \right)$ for each participant $i$ in the UKB dataset, yielding a recalibrated 10-year risk prediction ${\hat{\text{risk}}}_{\text{recal},i}\left( 10 \right)$ using the relation:

${\hat{\text{risk}}}_{\text{recal},i}\left( 10 \right)=1- g^{-1}\left( \hat{\alpha}+\hat{\beta}g\left( 1-{\hat{\text{risk}}}_{\text{pred}, i}\left( 9 \right) \right) \right)$ (3)

**Estimation of reclassification and translation to 100,000 UK individuals**

We used the recalibrated predicted CVD risk ${\hat{\text{risk}}}_{\text{recal},i}\left( 10 \right)$ to estimate the reclassification of individuals, between risk categories used in clinical guidelines (e.g., <5%, 5-10%, ≥10% of 10-year CVD risk) [12, 13].

To express our findings in a more clinically accessible manner, we used the information observed in the reclassification tables to generalize our findings to the context of population screening. We modelled a hypothetical UK population of 100,000 individuals, with sex- and age-specific structure the same as that of the standard UK population (2017 mid-year population, <https://www.ons.gov.uk/>), and CVD incidence rates as observed in CPRD (**Table A in S2 Text**). We assumed that treatment with statins would reduce the risk of CVD by 20%. We assumed treatment were allocated to those: 1) estimated to be at high risk according to the recalibrated risk; 2) had history of diabetes; or 3) with LDL cholesterol levels of 190 mg/dL or greater.

**Examination of the assumptions made in recalibration process**

Our methods assume that the recalibration of predicted risks from models fitted to UKB participants gives appropriate approximation to the predicted risk distributions that would be obtained if the models were derived and applied directly in the CPRD population. By extension, we assumed that the proportions of cases and non-cases falling into clinically relevant risk categories were representative of those that would be seen in the CPRD population. Since few biomarkers and no genetic data are available in our CPRD dataset, we tested our recalibration approach using simpler prediction models involving only age, sex, smoking and diabetes status. We followed these steps:

1. A Cox model was fitted to UKB participants, stratified by sex, and using age, smoking status and diabetes as predictors to obtain 9-year risk estimates for all UKB participants (${\hat{\text{risk}}}_{\text{pred},i}^{\text{UKB}}\left( 9 \right)$)
2. Risk estimates ${\hat{\text{risk}}}_{\text{pred},i}^{\text{UKB}}\left( 9 \right)$ were recalibrated using the CPRD incidence rates as described above, giving recalibrated 10-year risk estimates for all UKB participants (${\hat{\text{risk}}}_{\text{recal},i}^{\text{UKB}}\left( 10 \right)$)
3. The same specification of Cox model in (1) was fitted to CPRD participants to obtain 10-year risk estimates for CPRD participants (${\hat{\text{risk}}}_{\text{pred},i}^{\text{CPRD}}\left( 10 \right)$)
4. The distributions of ${\hat{\text{risk}}}_{\text{recal},i}^{\text{UKB}}\left( 10 \right)$ and${\hat{\text{risk}}}_{\text{pred},i}^{\text{CPRD}}\left( 10 \right)$ were compared to confirm that the former is a reasonable approximation to the predicted risk distribution we would expect to see in the wider CPRD population (**Fig C in S3 Text** and **Fig D in S3 Text**)

Good agreement between age-specific risk predictions in CRPD (${\hat{\text{risk}}}_{\text{pred},i}^{\text{CPRD}}\left( 10 \right)$) and the medians from risk predictions in UKB after recalibration (${\hat{\text{risk}}}_{\text{recal},i}^{\text{UKB}}\left( 10 \right)$) indicated that the predicted risk distribution after recalibration in UKB was representative of the predicted risk distribution in a general primary care setting.

**Fig A in S3 Text: Analytic components and process of estimation of potential public health impact**

**
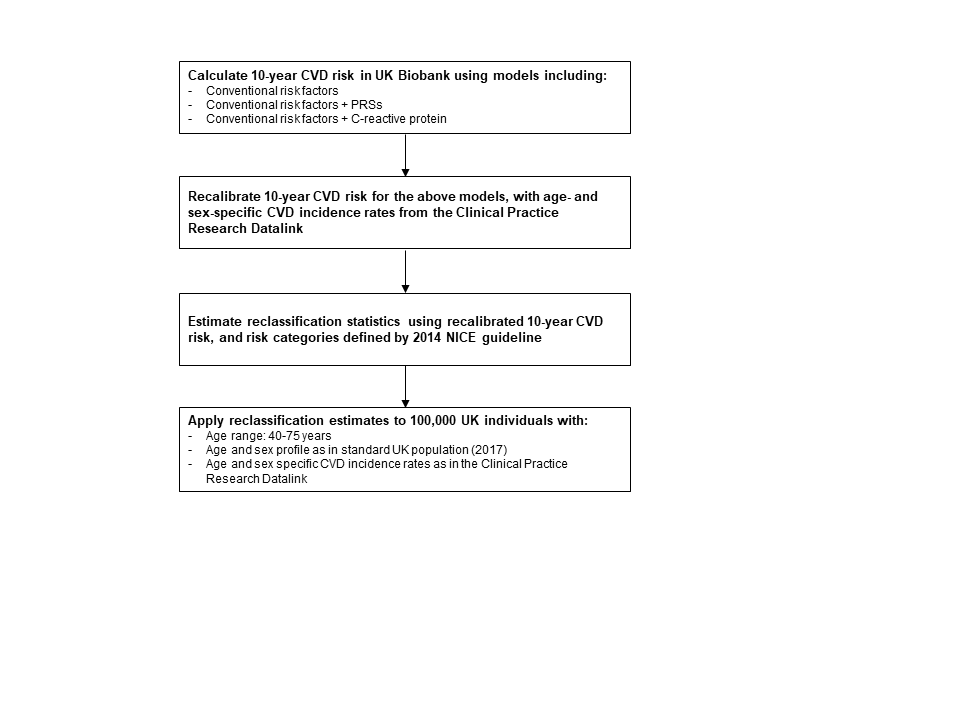
**

Conventional risk factors included information on age, sex, smoking status, history of diabetes, systolic blood pressure, total cholesterol and HDL cholesterol. Age- and sex-specific CVD incidence rates from the Clinical Practice Research Datalink were estimated among individuals without history of vascular diseases, and not on lipid-lowering treatment, at registration. Records (1st Apr, 2004 – 21 Oct, 2017) on about 2.1 million patients were used in the present analysis.

**Fig B in S3 Text: Comparison of sex-specific 5-year age-at-risk incidence rates of CVD between UKB and CPRD participants**

**

**

CVD, cardiovascular disease, including myocardial infarction, fatal coronary heart disease, and any stroke in both UKB and CPRD

**Fig C in S3 Text: Comparison of the predicted risk in UKB after recalibration and the predicted risk in CPRD, by sex and case status at 9 years since recruitment**

**

**

**Fig D in S3 Text: Density plots of the predicted cardiovascular risk for UK Biobank participants with and without recalibration to the targeted population**

**

**
