# Supplementary material for: Polygenic risk scores in cardiovascular risk prediction: A cohort study and modelling analyses
Source: PLoS Med. 2021 Jan 14;18(1):e1003498. doi: 10.1371/journal.pmed.1003498 (PMC7808664; doi:10.1371/journal.pmed.1003498)
Supplement: Attachment — Submitted filename: Responses_to_reviewers_20_May_2020.docx [file pmed.1003498.s030.docx]

**Responses to comments of editors and reviewers**

**Editors:**

1. Title- Please revise your title according to PLOS Medicine's style. Your title must be nondeclarative and not a question. It should begin with main concept if possible. "Effect of" should be used only if causality can be inferred, i.e., for an RCT. Please place the study design ("A randomized controlled trial," "A retrospective study," "A modelling study," etc.) in the subtitle (ie, after a colon).

**DONE. As suggested by the Editors, we have revised the title to be: “Polygenic risk scores in cardiovascular risk prediction: prospective cohort study and modelling analyses”.**

2. Author summary-At this stage, we ask that you include a short, non-technical Author Summary of your research to make findings accessible to a wide audience that includes both scientists and non-scientists. The Author Summary should immediately follow the Abstract in your revised manuscript. This text is subject to editorial change and should be distinct from the scientific abstract. Please see our author guidelines for more information: <https://journals.plos.org/plosmedicine/s/revising-your-manuscript#loc-author-summary>

**DONE.**

3. Abstract methods and findings- the last sentence should be a limitation of your study design

**DONE. It is now stated in the Abstract, Methods and findings, last sentence, that: ”Potential limitations of our study include its restriction to European ancestry participants and a lack of health economic evaluation”.**

4. Abstract background- please clearly state the aim of your study

**DONE. It is now stated in the Abstract, Background section that: “We aimed to quantify the potential advantage of adding information on PRSs to conventional risk factors in the primary prevention of CVD”.**

5. Abstract conclusions- please tone down in view of the “modest gains” in prediction accuracy

**DONE. The revised conclusions are now stating that: “Addition of PRSs to conventional risk factors provided modest improvement in prediction of first-onset CVD. Nevertheless, these moderate improvements could translate into meaningful clinical benefit if applied at scale and targeted to people at intermediate CVD risk”.**

6. Data availability- please provide details of how the data may be accessed, providing contact details, URLs or accession numbers as needed

**DONE.**

7. Page 3- please include a header for “Introduction”. **DONE.**
8. Reference square brackets must be placed before a full stop for example [2,5]. **DONE**
9. Methods- please introduce CHD on first view**. DONE.**
10. A table reference is missing on Page 9line 6**. DONE.**
11. Please complete the **TRIPOD checklist**, and include the completed checklist as Supporting Information. When completing the checklist, please use section and paragraph numbers, rather than page numbers. Please add the following statement, or similar, to the Methods: "This study is reported as per xxx guideline (S1 Checklist)."

**DONE.**

12. Overall the language needs to be toned down as this is an observational study**. DONE.**

**Reviewer #1:**

This study investigated the added values of polygenic risk scores in addition to conventional risk factors in prediction of cardiovascular disease. However, there are quite a few major issues needing attention.

1. Cox models were used throughout the study to predict CVD events. As the outcomes here were CVD events other than all cause mortality, there is a major issue of competing risk, which unfortunately was not addressed in the paper.

**DONE. We have now included additional analyse adjusting for competing risk. In the Methods section, page 7, lines 212-214 it is now stated that: “Sensitivity analyses included calculation of cumulative incidence of CVD outcomes based on the cause-specific hazards estimated from Cox regression, in the presence of competing risk from non-CVD deaths”. Furthermore, in the Results section, page 10, lines 291-296, it is now stated that: “Furthermore, similar results were observed in analyses using ….. competing risk models from non-CVD deaths (S8 Table)”.**

2. The authors developed the PRSs through 10-fold cross validation using the UK biobank data. However, there was no external validation (using independent external datasets) for the models, which is crucial for the validation of the developed models. Also, there is no calibration of the developed models. Could authors please read and follow the TRIPOD statement?

**DONE. We have clarified in the Methods section that PRS were developed using external weights from previous GWAS studies. On page 7, lines 190-201, it is now stated that: ”PRSs were previously constructed using a meta-score approach based on external summary statistics from the previous largest GWAS studies of CHD and stroke. Detailed information on PRSs derivation has been previously described, and publicly available and annotated at the PGS Catalog (www.pgscatalog.org) under accessions PGS000018 and PGS000039, respectively. The PRS for CHD comprised 1,743,179 variants where the mixing weights of component scores were estimated using 3000 participants in UKB. The PRS for ischaemic stroke included 2,759,740 variants where the mixing weights of component scores were estimated using 12,000 participants in UKB (including the 3000 participants mentioned above). Participants used in the training dataset were excluded from subsequent analysis. Previous analyses have not found evidence of overfitting, and independent replications have demonstrated consistent effect sizes”.**

**Furthermore, we have clarified in the Methods section, page 8, lines 221-225, that: “To avoid optimism, we applied an internal/external validation approach by validating within a subset (i.e., one study centre, or 10% randomly selected population in UKB) the prediction model derived from the remaining datasets. Results were then meta-analysed across all validation subsets, weighted by the number of events in that specific subset”. Results from this analysis are also presented in S8-9 Figures. Finally, a TRIPOD checklist check-list provided in the supplementary material.**

3. The prediction model containing conventional risk factors alone gave a C-index of 71% while the PRSs increased the C-index by 1% when adding to the model. Although this 1% is statistically significant, it is mainly because the huge sample size which will detect any tiny differences. Firstly this 1% increase hasn't gone through rigorous external validation, secondly the predictive model is not really convincing as hasn't adjusted for competing risk, and finally whole exercise of adding PRSs to the prediction model to increase the precision from 71% to 72% doesn't offer real practical benefits in clinical settings. It's more like a exploratory or association study to show the PRSs are of predictive value in predicting CVD outcomes, however this is already known.

**DONE. We have clarified in the Introduction, on page 4, lines 114-119 that: “The clinical utility of PRSs in CVD risk prediction is, however, uncertain. Previous analyses have generally focused only on coronary heart disease (CHD) rather than the composite outcome of CHD and stroke which is the focus of most primary prevention guidelines. Most previous PRS studies have also lacked modelling of clinical implications of initiating guideline-recommended interventions (e.g., statin therapy), meaning that it is has been difficult to judge the potential clinical gains of assessing PRSs“. Furthermore, as described above in response #1 and #2, we have now included analyses using competing risk and an internal/external validation approach.**

4. The whole excise of using CPRD data to show the benefits of the added value of PRSs is very difficult to follow and not convincing at all, because everything was based on assumptions especially for different projected outcomes. The argument should be based on the solid outcomes not some arbitrary and hypothetical scenarios.

**DONE. To help clarify the rationale and the approach of using external data from general population to model the implications of adding a PRS to CVD risk prediction in a primary-care setting we have modified the manuscript in several sections.**

**First, to clarify the study design, we have added to the main manuscript a flow-diagram (Figure 1) describing the different steps used in the analysis.**

**Second, in the Methods section, page 8, lines 232-237, it is now stated that: “We recalibrated risk prediction models derived in UKB to give 10-year risks that would be expected in a UK primary care setting using CPRD data, using methods previously described. This approach allowed more generalisable reclassification estimates than those directly derived from UK Biobank. Details are provided in S3 Text”.**

**Third, in the Results section, page 10, lines 297-304, we have clarified that: ”To assess the public health and clinical relevance of adding PRSs to conventional risk factors, we generalised our reclassification analyses to the context of a UK population eligible for risk assessment for CVD primary prevention. We used age and sex-specific incidence data from 2.1 million patients in the CPRD without previous CVD and not taking statin treatment to recalibrate risk models and achieve a predicted risk distribution as would be expected in this primary care population (S3 Text). We translated age- and sex-specific targeted assessment of PRSs to a population of 100,000 adults aged 40-75 years, assuming the age- and sex-structure of the current UK population, and CVD incidence rates observed in UK primary care”.**

**Finally, the revised S3 Text provides additional details about the methods and data used to for estimating public health impact.**

5. Figure 1 and 2 are a bit strange to put baseline characteristics and odd ratios together. It would be good to be straightforward and clear to have a baseline table to put UK biobank and CPRD data side by side, then have other tables for prediction models, validation, NPI, and etc.

**DONE. We have added a table of baseline characteristics for UK Biobank participants (Table 1) and a separate figure presenting hazard ratios (Figure 2).**

6. A bit confused and unclear on which models the authors worked on, predict stroke? predict CHD? but the CVD events were defined as stroke or CHD? Also, need to describe exactly which are the derivation and validation datasets, and any independent external validation datasets?

**DONE. We now clarified this in Method, page 6, lines 185-189, that: ”** **The primary outcome was a first CVD event, defined as CHD (i.e., myocardial infarction or fatal CHD) or any stroke. Secondary outcomes included each of CHD and stroke separately, and a combination of CHD, stroke, and cardiac revascularisation procedures (i.e., percutaneous transluminal coronary angioplasty [PTCA], and coronary artery bypass grafting [CABG]) (S1 Table)”. The approach used for internal / external validation is described above in response #2.**

**Reviewer #2:**

Sun et al. study the performance of two previously developed and validated polygenic risk scores for coronary heart disease and stroke. The authors assess the improvement in risk prediction on top of conventional risk factors using around 300,000 UK Biobank participants and estimate the clinical benefits by modeling the impact on data from a primary care setting.

The paper is well written and in line with several recent studies. The added value of this work comes from the recalibration of risk and modeling to a primary care setting in the UK.

1. As the authors are projecting the improvements into general benefits in the primary care setting, it is important that the performance of the scores is additionally assessed in other ancestries and not restricted to individuals of European ancestry.

**DONE. We agree that it is important to assess the performance of PRSs in other non-European ancestries. However, people of non-European ancestry make up a small proportion (<5%) of the UK Biobank. We have now stated the Discussion, page 13, lines 388, that: “Our study also had limitations. It was restricted to middle-aged European ancestry participants…”.**

2. The stroke PRS was derived from GWAS summary statistics of 19 phenotypes including stroke and coronary artery disease. What is the correlation between the stroke PRS and coronary heart disease PRS? In previous work the authors have combined polygenic risk scores in a metaGRS, why haven't they followed similar strategy here and created such score for CVD?

**DONE. We have now clarified in the Methods section, page 7, lines 190-205, the methodology used to derive the PRSs and the correlation between them: “We utilised separate PRSs for CHD and ischaemic stroke as two independent variables to predict the composite CVD outcome. PRSs were previously constructed using a meta-score approach based on external summary statistics from the previous largest GWAS studies of CHD and stroke. Detailed information on PRSs derivation has been previously described, and publicly available and annotated at the PGS Catalog (www.pgscatalog.org) under accessions PGS000018 and PGS000039, respectively. The PRS for CHD comprised 1,743,179 variants where the mixing weights of component scores were estimated using 3000 participants in UKB. The PRS for ischaemic stroke included 2,759,740 variants where the mixing weights of component scores were estimated using 12,000 participants in UKB (including the 3000 participants mentioned above). Participants used in the training dataset were excluded from subsequent analysis. Previous analyses have not found evidence of overfitting, and independent replications have demonstrated consistent effect sizes. The partial Pearson correlation coefficient between PRS for CHD and PRS for stroke was 0.32. In sensitivity analyses we replaced the PRS for ischaemic stroke with a PRS for all stroke, and used a single PRS for the composite CVD outcomes rather than using the combination of PRSs for CHD and ischaemic stroke as two independent variables.”.**

3. Both scores were derived and validated in the UK Biobank which raises some questions regarding external validity. This should be discussed. How would the score perform in a non-UK Biobank dataset?

**DONE. See response #2 to Reviewer #1.**

4. Use of antihypertensive medication is usually included with conventional risk factors and risk equations. Why is it not included here?

**DONE. We have included results from this additional analysis in the supplementary material. It is now stated on Results, page 10, lines 286-290, that: “The results of adding information on PRSs were broadly similar to those observed overall in analyses that included: information on body-mass index, family history of CVD, use of blood pressure-lowering treatment, or CRP in the prediction model (S4 Table and S7 Figure);…”.**

5. The authors use CRP for comparison with the PRS. How does the PRS perform on top of conventional risk factors that additionally include CRP?

**DONE. See response #4 to current Reviewer.**

**Reviewer #3:**

In this manuscript, Sun and colleagues investigate (a) whether the addition of polygenic risk scores for cardiovascular disease to a clinical model yields a change in risk classification at a population level; and (b) whether using this modified score to influence statin prescriptions is likely to yield a reduction in incident cardiovascular disease in a primary prevention population. These questions are timely and important in the field, coming at a moment when the PRS literature is in transition from demonstrating disease association to showing potential public health impact from implementation.

A substantial body of prior work, with important contributions from these authors, has demonstrated that polygenic risk scores can function as independent risk factors for incident cardiovascular disease (Inouye, M. et al. Genomic Risk Prediction of Coronary Artery Disease in 480,000 Adults: Implications for Primary Prevention. Journal of the American College of Cardiology 2018) and can improve 10-year risk classification when compared to clinical risk factors alone in various cohorts, including similar work in the UK Biobank that was published after this manuscript's submission (Abraham, G. et al. Genomic prediction of coronary heart disease. Eur Heart J 2016; Elliott, J. et al. Predictive Accuracy of a Polygenic Risk Score-Enhanced Prediction Model vs a Clinical Risk Score for Coronary Artery Disease. JAMA 2020). Although the primary dataset and some of the content is similar to the now-published Elliott et al manuscript, this manuscript extends prior work by asking how many incident CVD events might be prevented in the general UK population if statin prescribing patterns were changed by using a model that adds a polygenic score to classical risk factors. Because the costs (such as those due to genotyping and the increased number of statin prescriptions) were not addressed, some important questions raised by this manuscript are left unanswered. Some amount of the novelty was eclipsed by Elliott et al. However, the authors make key novel contributions (by assessing the potential impact of genomic screening to influence preventive statin prescriptions in a general UK population). Nevertheless, a comprehensive assessment would look not only at the potential benefits as described in this manuscript, but also at the cost and potential harms.

**DONE. See response #3 to Reviewer #1.**

Additional comments:

1. The description of the polygenic scores for CAD and stroke was insufficient to convey a complete understanding of how the scores were derived. The main text identified the source studies for the SNP weights (CARDIoGRAMplusC4D and MEGASTROKE, respectively) and indicated that plink 2.0 was used for genetic analysis. Supplementary Figure 4 indicated that "LD thinning" was performed in UK Biobank. "LD thinning" is not a named function in plink, and colloquially it can represent either (a) LD pruning on MAF or (b) LD clumping on GWAS P-value, both of which use r2 cutoffs and could therefore be the indicated method. Several other aspects of polygenic score creation in this manuscript were not clearly answered in the manuscript: most importantly, why were new scores created when polygenic scores using the same GWAS summary statistics have already been developed, tuned in a UK population, and publicly released (both by authors of this manuscript and others)? How was the r2 cutoff chosen and were other r2 cutoffs considered? The Data Availability section addresses UK Biobank and CPRD availability, but not the availability of the subsetted GWAS summary statistics necessary to compute the same scores in other populations; will those be made available? A more comprehensive treatment of the polygenic score will be important for permitting a complete evaluation, and for reproducibility. Alternatively, using polygenic scores that have already been derived and validated, such as scores previously published by coauthors of this manuscript, would sidestep this concern.

**DONE. See response #2 to Reviewer #2.**

2. The authors compare a CVD risk model whose inputs are clinical risk factors (the same as those that are used in the Pooled Cohorts Equation [PCE]) with a model that includes those clinical risk factors plus polygenic scores. It would be useful to also compare the "PCE+polygenic scores" model to the original PCE that uses the original risk factor weights (i.e., the PCE that is actually in clinical use). Doing so would permit a comparison with typical clinical practice, while the current comparison assesses a hypothetical reweighting of the PCE (which may be more optimally calibrated for the studied population, but which is not actually in clinical use) compared to a PCE+polygenic scores model.

**DONE. We have added the additional analyses in S7 Table. In the Results, page 10, line 291-294, it is now stated that: “Furthermore, similar results were observed in analyses using …. the Pooled Cohorts Equation (S7 Table) …”.**

3. The authors exclude individuals who are already taking statins from their main analysis, but importantly they provide a sensitivity analysis that includes those individuals. Those without pre-existing disease but who are taking statins for primary prevention form an important subgroup of individuals whose treatment decisions might be impacted by a PRS-informed cardiovascular disease risk score. Their exclusion can influence the apparent effectiveness of risk scores. For example, in the evaluation of the 2013 ACC Pooled Cohorts Equation, a sensitivity analysis that excluded the ~15% of MESA participants on statins substantially improved the C-statistic (by 0.0135 on average), showing that excluding statin users from risk calculators can have an important influence on model performance (see Goff et al, "2013 ACC/AHA Guideline on the Assessment of Cardiovascular Risk," 2014, Circulation, supplement pages 41-42). Here, the concern would be that excluding current statin-takers might differentially affect a risk score with a polygenic score compared to a risk score without a polygenic score. The authors' sensitivity analysis addresses that concern. It would be helpful to provide more comprehensive results from this sensitivity analysis (e.g., sensitivity, specificity, reclassification counts) rather than just showing the AUC change in Supplementary Figure 9.

**DONE. We have added the additional analyses in the supplementary material.** **In the Results, page 10, line 286-289, it is now stated that:** “**The results of adding information on PRSs were also similar in analyses that included: information on … participants receiving lipid-lowering treatment at baseline (S5 Table and S8 Figure);**

4. The likelihood ratio test (LRT) is the uniformly most powerful test comparing models with and without additional predictors. As such, while I believe that the authors have already chosen reasonable statistics to demonstrate clinical impact, for comprehensiveness it could be helpful to include a likelihood ratio test result to more precisely define the statistical improvement of adding the PRS (i.e., LRT of baseline vs baseline+PRS). In addition, a likelihood ratio test would permit a more concise answer to the question of whether the PRS adds information beyond C-reactive protein (i.e., LRT of baseline+CRP vs baseline+CRP+PRS). I raise this only as a suggestion for the authors' consideration.

**DONE. We have added the additional analyses in S4 Table**
