# Supplementary material for: Polygenic risk scores in cardiovascular risk prediction: A cohort study and modelling analyses
Source: PLoS Med. 2021 Jan 14;18(1):e1003498. doi: 10.1371/journal.pmed.1003498 (PMC7808664; doi:10.1371/journal.pmed.1003498)
Supplement: Attachment — Submitted filename: Responses_to_reviewers_2_Sept_2020v2.docx [file pmed.1003498.s031.docx]

**Responses to comments of reviewers (18-Aug-2020)**

**Reviewer #1:**

Thanks authors for their effort to improve the manuscript and I am satisfied with some of the responses. However, there are still remaining issues needing attention.

1. Regarding response #2, the authors didn't respond to my question on the calibration of the developed models. As described in the TRIPOD statement, the performance of clinical prediction models consists of both discrimination and calibration. So far in the paper the C-index and NPI are all about discrimination. However, there is no calibration of the models in the paper such as calibration plot/slope and other measures such as RMSE (root mean square error), therefore the performance evaluation is only half done.

**DONE.** **As suggested by the reviewer, we have now included additional analyses assessing calibration. In the Methods section, page 8, line 229-232, it is now stated:**

*“Calibration was assessed by comparing the observed and predicted risks across deciles of predicted risk and, and by calculating calibration slope, root mean square error, and the Greenwood-Nam-D’Agostino P value [13, 14, 33] using a ten-fold cross-validation approach to avoid optimism.”*

**In the Results section, page 9, lines 279-280, it is now also stated:**

*”Models including PRSs showed good calibration, with good agreement between the observed and predicted CVD risks (S11 Fig).”*

2. Regrading response #3, I am still not convinced about the clinical usefulness of adding the PRSs to conventional risk factors as only marginal improvement of around 1% in C-index was shown in the study which is also subject to complete independent external validation (eg, in another European country). Of course, if apply to large population, the 1% will always convert to some numbers but not sure whether it is cost effective and practical. Authors haven't touched the point of implication and practical usefulness of this marginal improvement by PRSs in the discussion, which is not adequate. Basically, one would wonder whether it's worth it to use all these genetic tests to gain only marginal improvement in prediction which doesn't seem very useful as compared to conventional risk factors.

**DONE. We have clarified text in multiple places in the revised manuscript to avoid giving the impression that this manuscript provides definitive evidence in favour of the population health usefulness of adding PRSs to conventional risk factors in CVD prevention. Instead, the revised text provides a more nuanced conclusion, highlighting the potential promise of PRSs in this setting (particularly when used at scale and in a targeted manner) but explicitly stating the uncertainties that remain. Hence:**

**• the Abstract conclusion (page 2, lines 59-61) now provides a more moderated conclusion, stating:**

*“Addition of PRSs to conventional risk factors modestly enhanced prediction of first-onset CVD, but this approach could still translate into meaningful population health benefit when applied at scale and when targeted among people at intermediate CVD risk.”*

**• the Discussion section (page 12, lines 347-349) now provides a specific illustration of the potential value of adding PRSs to conventional risk factors, stating:**

*“For example, if applied to the current UK population aged 40-75 years [37], additional use of PRSs could help prevent an additional ~21,000 CVD events over the next 10 years beyond assessment of conventional risk factors alone.”*

**• the Discussion section (page 13, lines 391-394) now directly addresses the need to evaluate the value of PRSs in different countries and in different ethnic groups, stating:**

*“We studied only middle-aged European ancestry participants in the UK, which limits the generalisability of our results. Hence, we (and others) are now addressing this gap by conducting studies of PRSs for CVD in multiple different ethnic groups, as well as in other countries.”*

**• the Discussion section (pages 13-14, lines 394-401) now directly acknowledges the need for health economic evaluations in future studies to address translational uncertainties, stating:**

*“Our study also lacked a health economic evaluation, which was beyond the scope of present analysis. We acknowledge the importance of health economic evaluations as part of future considerations to assess the clinical utility of PRSs for CVD prevention, noting that genome-wide array genotyping has a one-time cost (approximately £25 at current prices in the UK) and can be used to calculate PRS for CVD, as well as for many other chronic diseases. In particular, future studies (including health economic evaluations) are needed to evaluate a range of different CVD screening strategies, including a “genome-first” approach that inverts the current “conventional risk factors first” approach to CVDs.”*

**Reviewer #2:**

The authors addressed all my concerns.
