# Supplementary material for: Polygenic risk scores in cardiovascular risk prediction: A cohort study and modelling analyses
Source: PLoS Med. 2021 Jan 14;18(1):e1003498. doi: 10.1371/journal.pmed.1003498 (PMC7808664; doi:10.1371/journal.pmed.1003498)
Supplement: Attachment — Submitted filename: Responses_to_editors_30_Nov_2020.docx [file pmed.1003498.s032.docx]

**Responses to requests from Editors (17-Nov-2020)**

1. Title- please reword to "Polygenic risk scores in cardiovascular risk prediction: a modelling study"

**NOT DONE. To appropriately reflect the design of this study, we have retained the title: “Polygenic risk scores in cardiovascular risk prediction: a cohort study and modelling analyses”. Indeed, our analysis included two inter-correlated components: first, we estimated the incremental predictive values of addition of polygenic risk scores upon conventional risk factors using data from the UK Biobank, a large prospective cohort study; and second, to estimate the potential for disease prevention we modelled implications of initiating guideline recommended statin therapy in a primary-care setting using incidence rates from 2.1 million individuals from the Clinical Practice Research Datalink.**

2. Abstract- please add brief participant demographics

**DONE. As suggested by the Editors, we have now added a description of participants demographics in Abstract and stated as “Using data from UK Biobank on 306,654 individuals without a history of CVD and not on lipid-lowering treatments (age [SD]: 56.0 [8.0] years; females: 57%; median follow-up: 8.1 years), we calculated …”**

3. Abstract -Might be helpful to describe the C-index?

**DONE. We have now added a description of the C-index in Abstract (page 2) and stated as “The C-index, a measure of risk discrimination, was 0.710 (95% CI, 0.703-0.717) for a CVD prediction model containing conventional risk predictors alone.”**

4. The overall tone and language needs to be toned down to avoid overstating conclusions. Please add “our results indicate” or “modelling suggests” etc and adopt more cautious language. Conclusion- please add “our results suggest” or similar, to avoid overstating results. I’m not sure how this approach directly translates to application at scale, I suggest removing this part.

**DONE.**

5. Author summary- please add bullet points. **DONE.**
6. Model assumptions mentioned on page 8 should be briefly mentioned in the abstract as well. **DONE.**
7. Line 222, "to avoid optimism" should perhaps be "to critically evaluate this possibility ..." or similar? **DONE.**
8. Line 340-342 I suggest replacing this with a summary of the findings. **DONE.**
9. Line 348- 20,000 CVD events prevented-- needs to be toned down as this is not directly tested in your study. **DONE.**
10. Please remove financial information and data availability statements from the main text and add these to the article meta-data sections instead

**DONE.**

11. Did your study have a prespecified protocol or analysis plan? Please state this (either way) early in the Methods section. *If a prespecified analysis plan (from your funding proposal, IRB or other ethics committee submission, study protocol, or other planning document written before analyzing the data) was used in designing the study, please include the relevant prospectively written document with your revised manuscript as a Supporting Information file to be published alongside your study, and cite it in the Methods section. A legend for this file should be included at the end of your manuscript.*

**DONE.**

12. TRIPOD checklist- please use paragraphs and sections instead of page numbers as these are likely to change. **DONE.**
13. Please remove spaces from square brackets. **DONE.**
14. Please ensure p-values are reported to up to three decimal spaces. For eg Fig4 contains - p<0.0001. **DONE.**

**Responses to requests from Production Team (19-Nov-2020)**

**FIGURES and GRAPHICS:**

1. Please remove embedded figures from your manuscript file and ensure the most up to date versions of your figures are uploaded to our submission system as separate TIF or EPS files. Please do not remove the figure legends or captions from the manuscript. **DONE.**
2. Please ensure that all main figures are referenced in the manuscript as Fig 1, Fig 2, etc. (including capitalization), rather than Figure 1, Figure 2, etc. Please note, however, that the file names themselves must not include the space, i.e. Fig1.tif, Fig2.tif. Please ensure that all main figure files are clearly labelled as such in the File Inventory. **DONE.**

**METADATA:**

1. Please accurately complete the 'Competing Interests' section on our submission form, including any COIs declared by your co-authors. If you have no competing interests to declare, please add "The authors have declared that no competing interests exist". Otherwise please declare all competing interests beginning with the statement "I have read the journal's policy and the authors of this manuscript have the following competing interests:

**DONE.**

Your co-author, Dr. Kaptoge, has declared the following competing interest in relation to this manuscript. Please include it in the main competing interest statement in our system, sticking as closely as possible to Dr. Kaptoge's wording, using only their initials.

 "Grants to institution from: British Heart Foundation, UK Medical Research Council, UK National Institute of Health Research, Cambridge Biomedical Research Centre."

Your co-author, Dr. Burgess, has declared the following competing interest in relation to this manuscript. Please include it in the main competing interest statement in our system, sticking as closely as possible to Dr. Burgess's wording, using only their initials.

"SB is a paid statistical reviewer for PLOS Medicine."

Your co-author, Dr. Butterworth, has declared the following competing interest in relation to this manuscript. Please include it in the main competing interest statement in our system, sticking as closely as possible to Dr. Butterworth's wording, using only their initials.

"I have received grants outside of this work from AstraZeneca, Biogen, Bioverativ, Merck, Novartis and Sanofi, as well as personal fees from Novartis."

Your co-author, Dr. Danesh, has declared the following competing interest in relation to this manuscript. Please include it in the main competing interest statement in our system, sticking as closely as possible to Dr. Danesh's wording, using only their initials.

"John Danesh sits on the International Cardiovascular and Metabolic Advisory Board for Novartis (since 2010), the Steering Committee of UK Biobank (since 2011), the MRC International Advisory Group (ING) member, London (since 2013), the MRC High Throughput Science ‘Omics Panel Member, London (since 2013), the Scientific Advisory Committee for Sanofi (since 2013), the International Cardiovascular and Metabolism Research and Development Portfolio Committee for Novartis and the Astra Zeneca Genomics Advisory Board (2018)"

Please ensure only the authors' initials are included in the competing interest statement, not their full names or titles.

2. In accordance with PLOS' data policy, please ensure that your Data Availability Statement in the submission form clearly identifies how readers can access your data. Note that it will be typeset as it is written so please ensure it is in complete sentences and appears as you would like it to in the published version. For details on our data policy, please see <http://journals.plos.org/plosmedicine/s/data-availability>

**DONE.**

**ARTICLE FILE:**

1. Please indicate the corresponding author on the title page with an asterisk (*) and include the corresponding author's email address. Only an asterisk and the email address itself are required. **DONE.**
2. Please remove author degree titles from the byline of your manuscript file. **DONE.**
3. Please ensure that all author names are spelled correctly in the manuscript file and the submission form, including the use of a middle name/initials, and any special characters (accents, umlauts, etc.). Please place first/given names before last/family names. We ask that you ensure they match verbatim. Please be aware that correct spelling and formatting of author names in the article file is vital for accurate indexing in PubMed. **DONE.**
4. Please ensure that your Ethics Statement is available in the Methods section of your manuscript in its entirety, under the subheading "Ethics Statement". **DONE.**
5. We have noted that you have the following present in your manuscript: List of keywords/ Financial Disclosure/ Data Availability Statement. We will remove these section from your manuscript file as this information is automatically pulled from the submission form. Please make sure that the information in the submission form is correct.

**DONE.**

6. We do not publish any copyright or trademark symbols that usually accompany proprietary names, eg ©, ® or TM (e.g. next to drug or reagent names). Therefore please remove all instances of trademark/copyright symbols throughout the text, including © on page 15. **DONE.**

**REFERENCES:**

1. Please make sure all references are cited in ascending numerical order in the text. **DONE.**

**SUPPORTING INFORMATION:**

1. Please upload your Supporting Information items individually, rather than in a single file, as you refer to them individually in the text. If you would like to retain the single file please rename internal items (e.g. tables) as A, B, C etc. Please also provide a legend for each under the heading Supporting Information. Supporting Information files should be less than 10 MB each.  Please edit any citation of internal items within your Supporting Information files to using letters rather than numbers, e.g. “Table A in S1 Text”. Please ensure these changes are made throughout the manuscript and within the Supporting Information files themselves. **DONE.**
2. Please ensure that all Supporting Information files use the following format verbatim throughout the manuscript (including legends and all in-text citations): S1 Fig, S1 Table, S1 Text, S1 Data, etc. Each file type should start at 1 and increase numerically. Please note, however, that the file names themselves must use an underscore rather than a space, i.e. S1_Fig.tif, S1_Table.xlsx, S1_Text.doc, S1_Data.xlsx, S1_TRIPOD_Checklist.docx, etc. **DONE.**
3. Please ensure that each supporting information file has a legend at the end of the manuscript file, after the Reference list. **DONE.**
4. Please note that your submission cannot contain any references to generic "supporting information" files and instead must refer to specific files using our naming convention (S1 Fig, S1 Table, S1 Text, S1 Data, etc.). **DONE.**
5. Please replace the page numbers in your TRIPOD statement with paragraph numbers per section (e.g. "Methods, paragraph 1") since the page numbers of the final published paper may be different from the page numbers in the current manuscript. **DONE.**

Before resubmitting your paper, please ensure that all files referenced in the manuscript are present in the File Inventory, as missing files will result in further delays. Please also ensure that all file names adhere to our naming conventions and that all in-text citations and supporting information file legends/captions do as well.
